# Empty Follicle Syndrome Following GnRHa Trigger in PCOS Patients Undergoing IVF Cycles

**Published:** 2018

**Authors:** Krishna Deepika, Davuluri Sindhuma, Bijlani Kiran, Nair Ravishankar, Praneesh Gautham, Rao Kamini

**Affiliations:** - Department of Reproductive Medicine, Milann, The Fertility Center, A Unit of BACC Health care pvt Ltd, Karnataka, India

**Keywords:** GnRH Antagonist, Empty follicle syndrome, Gonadotropin releasing hormone agonist trigger, Polycystic ovary syndrome, Rescue trigger

## Abstract

**Background:**

The objective of this study was to analyze the incidence and the underlying mechanisms of empty follicle syndrome (EFS) occurring in gonadotropin releasing hormone agonist (GnRHa) triggered in *in vitro* fertilization (IVF) cycles with GnRH antagonist protocol in women with polycystic ovary syndrome (PCOS) of Indian origin. The study also intended to evaluate the cycle outcome following a rescue trigger.

**Methods:**

Retrospective cohort analysis of data was extracted from the hospital database of 271 PCOS patients who underwent IVF in antagonist protocol triggered with GnRHa from August 2014 to December 2016. All cases with failure to obtain oocytes following retrieval were analyzed. Continuous variables were expressed as mean±SD using t-test and Chi-squared test for categorical variables. A p<0.05 was considered statistically significant.

**Results:**

Incidence of EFS following GnRHa trigger was found to be 3.3%. False empty follicle syndrome (FEFS) accounted for majority of the cases (8/9=88.8%). Of the nine EFS, six cases were salvaged with a rescue trigger, resulted in transfer of reasonably good quality embryos in a frozen-thawed embryo replacement cycle achieving clinical pregnancy in three cases (3/6=50%).

**Conclusion:**

Our experience with EFS cases following GnRHa, albeit small, given the rarity of its occurrence, suggests that majority of EFS are of false forms and can be effectively salvaged which results in reasonably favorable outcome.

## Introduction

Empty follicle syndrome (EFS), still an enigmatic syndrome, is undeniably annoying as it raises stress and concern for both the treating physician and the patient (1). EFS is defined as a condition in which no oocytes are obtained from mature follicles following ovarian stimulation in an assisted reproductive technology (ART) cycle with apparently normal follicular growth and steroidogenesis. The incidence of this syndrome has been reported as 0.6–7.0% (2, 3). As EFS cannot be predicted by the pattern of ovarian response to stimulation, either by sonography or hormonal parameters, the diagnosis is retrospective. The ovulation trigger, crucial for the achievement of meiotic maturity and developmental competence, with its effect on loosening the cumulus oocyte complex (COC) from the follicular wall, may play a role in the pathophysiology of EFS. Human chorionic gonadotropin (hCG), due to its similarities with luteinizing hormone (LH), has been used to induce final oocyte maturation after ovarian stimulation in all IVF cycles. However, hCG due to its longer half-life and prolonged luteotrophic action can result in ovarian hyper stimulation syndrome (OHSS), more so in PCOS and hyper-responders (4). During recent years, with GnRH antagonist protocol (GnRHa) becoming more widely used, gonadotropin releasing hormone agonist (GnRHa), as a trigger has gained popularity, alternative to hCG in the prevention of OHSS in PCOS patients undergoing IVF cycles (4).

It has been proven that GnRHa effectively stimulates final oocyte maturation in GnRHa cycles (4–6). However, the GnRHa induced surge differs with that of natural cycle LH surge in both the duration and profile (6). The LH surge occurring in natural cycle lasts for 48 *hr* and comprises three phases: a rapidly ascending phase and plateau phase each lasting for 14 *hr* and a descending phase of 20 *hr* (7). However, the GnRHa induced LH surge lasts for shorter duration (24–36 *hr*) and comprises only two phases: a short ascending limb (∼ 4 *hr*) and a long descending limb (∼ 20 *hr*) (6). On the contrary, hCG induces an LH-like activity lasting for 8–9 days due to its high biologic activity (7). hCG acts directly on the LH receptors in the ovary, while GnRHa acts indirectly through the pituitary, releasing endogenous gonadotropins. Thus, EFS following GnRHa trigger may not be the same as that resulting from hCG trigger. Majority of the studies published so far have evaluated the causative factors of EFS following hCG trigger, with not much data available for GnRHa triggered cycles. The present study aimed to analyze the incidence, the underlying mechanisms of EFS in GnRHa triggered IVF cycles in PCOS. In addition, an attempt was made to evaluate the cycle outcome following a rescue protocol with a second trigger.

## Methods

### Study design:

The study is a retrospective cohort analysis of data of 364 GnRHa triggered IVF cycles in GnRHa protocol between August 2014 (when GnRH agonist was introduced as a trigger in PCOS patients for prevention of OHSS at our hospital) to December 2016. After applying inclusion and exclusion criteria to the data, 271 PCOS individuals triggered with GnRHa were found to be eligible. For eligible participants, data on ovarian stimulation, clinical and embryological outcomes were reviewed, with all cases of failure to retrieve any oocyte, despite the presence of optimal dominant and intermediate follicles and next their subsequent outcome following rescue trigger was analyzed.

### Study population:

Inclusion criteria were: (i) GnRHa triggered IVF cycles in PCOS, defined as per the ESHRE/ASRM (2003) Rotterdam criteria (8), (ii) Age of 21–37 years, (iii) D2/3 serum FSH concentration of<10.0 *IU/L*, (iv) Body mass index (BMI) of >18 and <30 *kg/m*^2^, (v) Presence of both ovaries, (vi) Indication for IVF/ICSI, and (vii) Stimulation in GnRHa protocol and one stimulation cycle for each patient.

Exclusion criteria were: (i) IVF cycles with evidence of premature ovulation, (ii) Patients with hypogonadotropic hypogonadism, (iii) PCOS triggered with human chorionic gonadotropin (hCG)/dual trigger (GnRHa+hCG), (iv) Donor cycles triggered with GnRHa trigger, (v)≥2 failed IVF cycles, and (vi) Surgical retrieval of sperm.

### Ovarian stimulation:

All PCOS patients were pretreated with oral contraceptive pill in the previous cycle. Day 2/3 follicle stimulating hormone (FSH), luteinizing hormone (LH), estradiol (E2), progesterone (P4), anti-mullerian hormone (AMH), and baseline transvaginal scan (TVS) were performed. Individualized controlled ovarian stimulation was started with recombinant follicle stimulating hormone (rFSH) (Recagon, Organon) and starting dose ranged from 112.5 to 175 *IU* daily. Follicular development was monitored by transvaginal sonography (TVS) using a 4–8 *MHz* vaginal probe (Aloka, Prosound 6) and serum E2 levels; the dose of gonadotropins was adjusted accordingly and if necessary, human menopausal gonadotropin (HMG, Menopur, Ferring) was added. Flexible multiple dose protocol was followed with GnRH antagonist and Ganirelix (Orgalutran, Organon) 0.25 *mg/day* subcutaneous (s.c) was added when the leading follicle was ≥14 *mm* and/or serum estradiol concentration was ≥300 *pg/ml* and continued till the day of trigger. On the day of trigger, serum E2, LH and P4 concentrations were measured. Final oocyte maturation was triggered with 2 ampoules of 0.1 *mg* of subcutaneous Triptorelin (Decapeptyl, Ferring) when three leading follicles achieved ≥17 *mm* diameter.

After September 2015, all GnRHa triggered cycles after 12 *hr* of trigger besides serum levels of LH (LH12) and P4 were measured. However, based on the post-trigger values, no intervention was taken as there was no certainty about the method of interpreting these measures. Transvaginal ultrasound-guided oocyte pick-up (OPU) was performed 35 *hr* post-trigger using single lumen oocyte retrieval needle under intravenous sedation. Before starting the OPU, adequacy of aspiration suction pressure and proper functioning of the pump apparatus were confirmed. If no oocytes were recovered from the first several follicles aspirated, the retrieval was aborted based on the assumption that the trigger injection had failed. The number of follicles aspirated before abandoning the retrieval attempt in these cases varied mostly from six to eight, depending on the judgment of the retrieval physician. EFS was taken as failure to retrieve any oocyte, after thorough aspiration and flushing of six-eight follicles (at least 3 dominant follicles ≥17 *mm* and 3 intermediate follicles between 14–16 *mm* in mean diameter). An attempt was made to rescue the cycle with recombinant hCG (rhCG), (Ovitrelle, Serono) 250 *μcg* s.c and a repeat oocyte retrieval was performed 35 *hr* later. Following the second retrieval, if oocytes were obtained, a freeze-all strategy of the embryos was employed. Post oocyte retrieval, an assessment for symptoms and signs of OHSS, was performed on day 4 and 7. However, patients were advised to present themselves at any point of time, in case of symptoms such as abdominal distension/pain, nausea, vomiting, diarrhea or difficulty in breathing. Transvaginal sonography was done to assess the ovarian size, free fluid in pouch of Douglas, paracolic gutters, Morrison’s pouch, pleura and a blood sample was collected to measure estradiol level and to detect hemoconcentration (hematocrit of 45%).

### Cryopreservation, thawing and frozen embryo transfer:

In all cases, intracytoplasmic sperm injection (ICSI) was performed as per the standard operating procedure of the hospital. Fertilization was checked 18 *hr* post ICSI by the appearance of two pronuclei. Cleavage stage embryos and blastocysts were graded as per the Istanbul consensus (9). Cleavage embryos as Grade 1(Good) had <10% fragmentation, stage-specific cell size and no multinucleation. Grade 2(Fair) had 10–25% fragmentation, stage-specific cell size for majority of cells and no evidence of multinucleation. Grade 3(Poor) had severe fragmentation (>25%), not stage-specific cell-size and evidence of multinucleation. As a hospital policy, all embryos on day 3 were vitrified by open system using cyrolock with 15% ethylene glycol, 15% dimethyl sulphoxide (DMSO) and 0.5 *mol/L* sucrose as cryoprotectants (Sage vitrification kit, Origio). On the day of transfer, the embryos were thawed using 1.0 *M* sucrose (Sage thawing kit, Origio). Following thawing, blastomere survival of ≥50% (with clear cellular boundaries and no degeneration) was identified as a live embryo. Depending on the number and grade of viable embryos following thawing, they were either transferred as cleavage embryos or cultured to blastocysts. Blastocysts were graded as 1-Early; 2-Blastocyst; 3-Expanded; 4-Hatched/ hatching; Inner cell mass: 1(Good)-prominent, easily discernible, with many cells that are compacted and tightly adhered together; 2(Fair)- easily discernible, with many cells that are loosely grouped together; 3(Poor)-difficult to discern, with few cells; Trophectoderm:1(Good)-many cells forming a cohesive epithelium; 2(Fair)-few cells forming a loose epithelium; 3(Poor)-very few cells.

All frozen embryo transfer (FET) cycles were performed in an artificial cycle with a daily dose of orally administered 6 *mg* of estradiol (Progynova; Zydus Cadila, German Remedies). When the endometrium evaluated by TVS was >8 *mm* with triple layer morphology, it was considered mature. This was followed by endometrial priming with 3 days of injectable progesterone (gestone 50 *mg*; Ferring) for cleavage embryos and 5 days for blastocysts. If the endometrial thickness was <7 *mm* on day 9, Oestrogel (Besins, Belgium) was added and the dose of estradiol was increased to 12 *mg*. If the endometrial thickness remained less than 7 *mm* in spite of additional estrogen supplementation, the cycle was cancelled. The maximum number of embryos transferred per FET cycle was three in cleavage embryos and two in blastocyst stage. The transfer was performed under ultrasound guidance using Cooks catheter (KJETS-7017-SIVF, Cook Medical, Sydney IVF). Luteal phase supplementation was continued with vaginal micronized progesterone (Susten, Sun pharma) and estradiol for 14 days and till 10 weeks of gestation, when clinical pregnancy was achieved.

### Immunoassay of hormones:

Serum levels of FSH, LH, E2 and P4 were assayed using an automated electro-chemiluminescent immunoassay system (Roche Cobas e411 analyzer). Assay sensitivity for FSH was 0.1 *mIU/ml* and LH was 0.1 *mIU/ml*. Linearity for FSH and LH was 200 *mIU/ml*. The minimum detection limit of E2 was 5.0 *pg/ml* and linearity up to 4300.0 *pg/ml*. AMH was measured using generation 2 ELISA kit (Beckman Coulter, Sensitivity: 0.08 *ng/ml*, Linearity: 0.16–22.5 *ng/ml*).

### Statistical analysis:

Data was analyzed using Statistical Package for Social Sciences version 16.0 (SPSS, USA). Continuous variables are expressed as mean values with standard deviation and categorical variables as number (%) across EFS and non-EFS population. The significance level between the observed mean values was calculated using t-test and Chi-squared test for categorical variables. A p<0.05 was considered statistically significant.

## Results

Table 1 presents the baseline and demographic variables of EFS and non-EFS in PCOS population triggered with GnRHa. The age, BMI, parity, cause and duration of infertility were similar in both groups. The stimulation characteristics are presented in table 2. As shown, there is no significant difference with respect to D2 FSH, AFC and AMH. However, significantly higher dosage of gonadotropins and prolonged stimulation was observed in the EFS group. Significantly higher number of intermediate follicles (14–16 *mm*), peak estradiol and progesterone levels were observed in the EFS population.

**Table 1.** Baseline characteristics of the study population

**Variables**	**Non-EFS (n=262)**	**EFS (n=9)**	**p-value**
**Age (years)**	29.33±3.64	30.1±2.08	0.53
**Primary infertility n (%)**	195(74.4)	7 (77.7)	0.82
**Secondary infertility n (%)**	67 (25.6)	2 (22.2)	0.81
**Duration of infertility (years)**	6.1±2.5	6.2±2.3	0.9
**BMI (*kg/m*^2^)**	24.3±4.31	22.8±3.91	0.30
**Irregular menstrual cycles n (%)**	173(66)	7(77.7)	0.46
**Clinical Hyper-androgenemia n (%)**	94(35.9)	4(44.4)	0.60
**PCOS with tubal factor infertility n (%)**	84(32)	2 (22.2)	0.53
**PCOS with male factor infertility n (%)**	139(53)	4(44.4)	0.61
**PCOS with endometriosis n (%)**	13(4.9)	1(11.1)	0.4
**Anovulatory infertility n (%)**	26 (9.9)	2 (22.2)	0.23

Values are expressed as mean±SD (95% CI) and n (%). SD=Standard deviation; P<0.05= statistically significant

**Table 2.** Stimulation cycle characteristics of the study population

**Variables**	**Non-EFS (n= 262)**	**EFS (n=9)**	**p-value**
**Day 2 FSH (*IU/L*)**	5.18± 1.42	4.68 ± 2.17	0.3
**AFC**	25.25 ± 6.98	23.7 ± 5.65	0.51
**AMH (*ng/ml*)**	5.79 ± 3.30	6.03 ± 2.20	0.82
**Duration of stimulation (days)**	10±1.2	11.9 ± 1.75	< 0.0001
**Dosage of gonadotropins (*IU*)**	1845±707	2473.8 ± 1748.2	0.01
**Dominant follicles >17 (*mm*) on the day of trigger**	12.7±4.3	10.5 ± 3.81	0.13
**Intermediate follicles 14–16 (*mm*) on the day of trigger**	9.9±3.3	12.9±3.4	0.007
**Peak E2 (*pg/ml*)**	4978.1±1451	5996.9 ± 1584	0.04
**Peak P4 (*ng/ml*)**	2.1±1.1	2.9± 1.2	0.03
**12 *hr* post-trigger LH (*IU/L*)**	44.8±21.3	14.84±10.65	0.0001
**12 *hr* post-trigger P4 (*ng/ml*)**	14±6.92	5.32 ± 4.56	0.0002

Values are expressed as mean±SD (95% CI). SD=Standard deviation; P<0.05= statistically significant. E2-estradiol, P4-progesterone

Table 3 illustrates the outcome of EFS cases following rescue trigger. Three EFS cases (case 1, 5 and 6) did not have embryos for transfer as a result of poor quality embryos due to poor quality oocytes [cytoplasmic (dense granular cytoplasm and aggregation of smooth endoplasmic reticulum) and extra-cytoplasmic abnormalities (large perivitelline space with debris and fragmented polar bodies)]. In four EFS cases (1, 4, 6 and 9), LH12 value was observed to be less than 15 *IU/ml*. Although two cases (5 and 7) had a 12 *hr* post-trigger LH of >15 *IU/ml* and P4 of >3.5 *ng/ml*, yet no oocytes were retrieved in the 1st OPU. Subject eight’s LH12 was >15 *IU/ml* (17.1 *IU/ml*), yet no oocytes were recovered. However, this subject was observed to have a low post-trigger P4 value (P4=1.7 *ng/ml*). Of interest, 2 EFS rescued subjects who failed to conceive in the 1st FET had good quality embryos still frozen and are yet to come for transfer.

**Table 3.** Cycle outcome of EFS cases following rescue trigger

**EFS cases**	**LH (*IU/L*)**	**P4 (*ng/ml*)**	**Rescue trigger**	**Number oocytes**	**Number of MII**	**Number fertilized**	**Number and embryos grade**	**Number and grade FET**	**Outcome**
**1**	10.9	4.2	rhCG ^*^	4	2	2	1-6CG3 (PQE¥)	--	Lost for follow up
**2**	NA	NA	rhCG	6	4	3	2-8CG1^•^ +1-8CG2^••^	2-8CG1	Blighted ovum
**3**	NA	NA	rhCG	7	6	6	6-8CG2	#2-Blast(2-1-1;1-1-2)	Severe OHSS Miscarriage at 8weeks
**4**	10.2	2.3	GnRHa^**^	10	7	5	4-8CG1	#2-Blast(3-1-1;1-1-2)	Ongoing pregnancy
**5**	34.5	9.8	rhCG	0	0	--	--	--	--
**6**	8.1	6.3	rhCG	4	2	2	Cleavage arrest	--	--
**7**	19.71	12.3	rhCG	8	6	5	5-8CG2	#1-Blast(2-1-1)	Failed to conceive
**8**	17.3	1.7	rhCG	9	7	6	5-8CG1	3-8CG1	Moderate OHSS Failed to conceive Has 2-8CG1 frozen
**9**	7.2	1	rhCG	8	6	6	6-8CG1	3-8CG1	Severe OHSS Failed to Conceive Has 3-8CG1 frozen

MII-mature oocyte; NA-Not available;

* Recombinant hCG (rhCG), 250 *mcg*;

** Gonadotropin-releasing hormone agonist, 0.2 *mg*;

• 8CG1-8 celled grade 1 embryos;

•• 8CG2-8 celled grade 2 embryos ¥PQE-poor quality embryos;

# blastocysts obtained after culturing cleavage embryos *in vitro* for 2days

## Discussion

EFS, though an uncommon complication of IVF, leading to cycle cancellation, is frustrating for patients mentally, socially and economically. The first report of EFS was by Coulam et al. in 1986 (10) and subsequently, most of the cases reported have been in cycles occurring after hCG trigger. GnRHa has emerged as the trigger of choice in PCOS, hyper-responders and donors, as it virtually eliminates or reduces the risk of developing OHSS because of its short half-life (60–120 *min*), limits the production of vascular endothelial growth factor, the key mediator of OHSS (11, 12). There have been only few case-reports describing EFS after GnRHa trigger in OHSS risk patients with not much of literature available. Hence, our study aimed to analyze the occurrence, the underlying etiopathophysiology and also the prevention of EFS following GnRHa trigger in PCOS patients of Asian origin undergoing IVF cycles in antagonist protocol. Additionally, the cycle outcome following rescue trigger was analyzed.

An EFS incidence of 3.3 (9/271) % following GnRHa trigger was reported in this paper. However, the sample size was comparatively smaller, as the usage of GnRHa as triggering agent in PCOS to prevent OHSS was implemented from 2014. The incidence of EFS following hCG trigger as reported in various studies, was 0.5–2% (13), 0.6–7% (14), and 2–7% (15), of IVF cycles. Our analysis shows that the incidence of EFS following GnRHa trigger seems to be almost similar to that occurring in hCG triggered cycles. Following GnRHa trigger, Castillo et al. (16) reported an EFS incidence of 3.5%, which is similar to that reported in our study.

The exact etiopathophysiology of EFS is still not understood clearly. It has been postulated that genuine EFS (GEFS) and false EFS (FEFS) are the two main mechanisms to explain failure to obtain oocytes following hCG trigger (17). Our study reports the incidence of FEFS following GnRHa trigger to be higher (88.9%), whilst GEFS accounted for 11.1%, similar to that reported following hCG trigger being 67% and 33%, respectively, indicating a relatively smaller risk of having GEFS (17). GEFS is related to intrinsic ovarian dysfunction and FEFS to drug related problem. The same may be applicable to GnRHa trigger, although the two triggers differ in terms of molecular structure, site and mechanism of action. Final oocyte maturation with GnRH agonist is applicable in stimulation cycles, where the pituitary gland remains responsive to GnRH agonist as it acts through initial flare-up effect, releasing LH and FSH (18). Thus, the fault may be an inability of the pituitary to release gonadotropins, or a failure in one of the mediators/receptors on the ovary. The ability to predict EFS following hCG or GnRHa trigger is clinically useful to determine whether to proceed with or cancel the oocyte retrieval and also to differentiate between GEFS and FEFS. Following hCG trigger, optimal levels of hCG concord with its correct administration, although the so called “optimal” levels are not clearly defined, with values on the day of oocyte retrieval of 40 *IU/L* (17), 100 *IU/L* (19), and 98– 161 *IU/L* (20). Following GnRHa trigger, measurements of LH and progesterone at 8–12 *hr* would probably predict the efficacy of the trigger and EFS. Unfortunately, an appropriate cut-off post-trigger value that will be useful clinically in predicting EFS, has still not been clearly established as firstly, the LH level peaks at ∼4 *hr* after trigger, gradually declining over 24 *hr* and P4 levels increase substantially following OPU (5). And secondly, a single time point estimation of serum LH might not be useful due to its pulsatile nature and that the duration of LH surge is a better predictor of oocyte maturation, although its measurement is not clinically practical (21).

However, regarding a post-trigger LH value at 8–12 *hr* of ≤15 *IU/ml* following GnRHa, the probability of EFS was 18.8% and it was unlikely for EFS to occur when it was ≥15 *IU/ml*, as elucidated by Kummer et al. (21). This post-trigger LH cut-off value of 15 *IU/ml* was found to have a sensitivity of 97.4% and specificity of 100% for predicting EFS. In addition, all EFS cases had post-trigger progesterone levels of ≤3.5 *ng/ml*, although these assessments were based on few patients manifesting with the incident. Further, it was found that an LH threshold of 8 *IU/L* was low enough to ensure the trigger had been administered around 36 *hr* prior, rather than 12 *hr*, even when the P4 value was high enough to support both the timings (22, 23). However, Shapiro et al. (24) observed a 12 *hr* post-trigger LH concentration of <52 *IU/L* as suboptimal, causing a modest reduction and a level <12 *IU/L* resulted in a dramatic reduction in oocyte yield and maturity. In our study, 12 *hr* post-trigger LH and P4 were significantly lower in the EFS population compared to the non-EFS population (Table 2). Surprisingly, in two EFS cases (5 and 7), although LH12 >15 *IU/ml* was observed, yet no oocytes were retrieved in the 1st OPU, probably because the LH12 <50 *IU/ml*. One might speculate that the minimal effective serum LH levels, 12 *hr* posttrigger, seem to be 12–15 *IU/L*, while optimal efficacy for a successful oocyte yield might be achieved when serum LH levels exceed about 50 *IU/L* (25). Thus, this suggests the cases with borderline range of relatively low post-trigger LH and P4 concentrations where there is a small likelihood of EFS and clinicians need to be cognizant of this possibility. A low or borderline post-trigger LH with an appropriately elevated post-trigger P4 concentration still could result in successful oocyte retrieval. Conversely, low post-trigger P4 concentration associated with LH concentration >15 *mIU/ml* might suggest suboptimal oocyte recovery (24).

Only one subject (1/9=11.1%), case 5, with 10 dominant follicles ≥17 *mm* and 12 intermediate follicles between 14–16 *mm* on the day of trigger, peak E2- 6779 *ng/ml*, with a 12 *hr* post-trigger LH and P4 values of 34.5 *IU/ml* and 9.8 *ng/ml*, respectively, failed to yield any oocytes, following rescue trigger with hCG. Presumably, this was a case of GEFS, probably attributable to dysfunctional folliculogenesis, as PCOS is commonly associated with it. Various hypotheses for occurrence of GEFS after hCG trigger include [1] Dysfunctional folliculogenesis (15), [2] Faulty functioning of granulosa cells (26), [3] Defective oocyte development and maturation (27), [4] Follicles need for a longer duration of exposure to the trigger, to undergo cumulus expansion and separation from the follicular wall (13), [5] Poor ovarian reserve with AMH levels ≤0.5 *ng/mL*, probably due to impaired folliculogenesis (28), [6] Genetic factors (27) of (a) LH/hCG receptor mutations and (b) altered expression of genes involved in cumulus expansion, cellular processes and apoptosis, and [7] Advanced ovarian ageing (15). It is, however, plausible that the same factors may be causative of GEFS after GnRHa trigger and occur in the presence of a normal endogenous LH and/ or progesterone rise. Such cases are unlikely to respond to rescue hCG protocol.

On the contrary, FEFS after GnRHa trigger occurs following a failure of induction of optimal endogenous LH surge and/or progesterone rise. In fact, empty follicle syndrome, is a misnomer for what often results from failed injection of the ovulatory trigger (17). Majority of EFS cases (67%) following hCG trigger, as reported by Stevenson et al. (17) in a systematic review were the false forms. In concordance, FEFS accounted for majority of the cases (8/9=88.8%) in our study. The potential reasons can be [1] Human errors in timing, preparation or administration of the triggering drug (13, 29), [2] Problems with manufacturing, packaging or shelf life of the trigger (19), and [3] Abnormality in the *in vivo* biological activity of some batches of commercially available GnRHa (21).

As the pituitary gland is the site of action for GnRHa, any temporary or permanent dysfunctions of the hypothalamic-pituitary-ovarian (HPO) axis might not produce optimal flare, resulting in deficient final follicular maturation and EFS. This happens in WHO type I anovulation, hypogonadotropic hypogonadism (HH), characterized by endogenous low levels of FSH and LH. Here, the pituitary gland may be suppressed from the lack of endogenous GnRH stimulation and thus may not reliably respond to GnRHa administration. Subject 3 with irregular cycles, near normal FSH and LH, AMH-6.9 *ng/ml*, classical polycystic ovarian morphology (PCOM) on scan as defined by the Rotterdam criteria (8), was stimulated with rFSH 150 *IU* for the 1st four days. An assessment on the fifth day of stimulation showed no ovarian response, hence rFSH was changed to HMG, dose being increased to 225 *IU*. 2 days later, as no follicular growth was noticed, the dose was further increased to 300 *IU*. After 12 days of stimulation, with 7 dominant follicles and 15 intermediate follicles, peak E2-7215 *ng/ml*, GnRHa was administered and an OPU 35 *hr* later resulted in EFS. This subject was actually a case of hypothalamic amenorrhoea (HA) with PCOM, misdiagnosed as PCOS as per the 2003 Rotterdam criteria. Albeit infrequent, classical PCOM as well as increased ovarian stroma has been encountered in patients with hypothalamic amenorrhoea (HA/PCOM) (30). In the absence of biochemical or clinical hyperandrogenemia, these patients clearly did not meet the diagnostic criteria for PCOS set forth by the National Institutes of Health conference in 1989 (31). The PCOM on scan is due to an increase in the ovarian cytochrome P450c17α activity, although it may be masked by the suppressed hypothalamic-pituitary axis (30). Under gonadotropin stimulation, this may result in exaggerated ovarian response and hyperstimulation. Over time, depending on the current status of hypothalamic activity, these individuals may fluctuate between symptoms of HA and PCOS. In this state, these women should be managed as other women with HH and not as PCOS. Hence, one has to cautiously make a diagnosis, as GnRHa triggering in this group of patients will inevitably result in EFS.

In agreement with this concept, it can be hypothesized that subjects with low circulating levels of LH and FSH, due to excessive suppression of HPO axis, as in long agonist protocols or following prolonged usage of oral contraceptive [as encountered in subject 9] would also run the risk of EFS after GnRHa trigger. This subject, prior to IVF had received combined oral contraceptive pills for three months and was stimulated with a dosage of 3,750 *IU* of gonadotropins for 12 days. Her E2 and P4 on the day of trigger were 7,680 *pg/ml* and 2.91 *ng/ml*, respectively and were triggered with GnRHa. Although 12 *hr* post-trigger LH and P4 were comparatively lower, no intervention was taken based on these values as optimal levels were still not defined. A rescue hCG trigger yielded 8 oocytes resulting in 6–8CG1 embryos. Retrospectively, looking into the cause of trigger failure and EFS, low values of LH on day 2 (0.9 *IU/L*) and on the trigger day (0.3 *IU/L*) were found and its importance was not realized till the mishap happened. Additionally, comparatively lower post-trigger values should have alerted us for intervention at that point of time rather than proceeding for OPU and encountering EFS. Pre-treatment with oral contraceptives for 3 months prior to IVF could have resulted in temporary HPO axis dysfunction, hence the trigger failure and EFS. In a retrospective cohort study by Meyer et al. (32), individuals who had lower FSH (<0.1 *vs*. 3.48) and LH (<0.1 *vs*. 2.51) levels on day 2 of cycle, lower LH (0.109 *vs*. 0.596) on the day of trigger were more likely to have GnRHa trigger failure. Supporting evidence for this was by Chang et al. (33), who identified prolonged stimulation and high total dosage of gonadotropin (>3,800 *IU*), as additional risk factors for trigger failure. Hence, to avoid this complication of trigger failure and resultant EFS, appropriate selection of patients and identification of risk factors are important.

Furthermore, patients who could be anticipated to have EFS, are those with GnRH receptor mutations (34), polymorphism (35) and variant LH β gene polymorphism, more so, in the homozygous form, resulting in a less bioactive LH molecule (36). The LH stimulation induces a transient sequential expression of epidermal growth factors such as amphiregulin, epiregulin and betacellulin. These growth factors induce expression of prostaglandin synthase 2, tumour necrosis factor alphainduced protein and hyaluronan synthase 2, which are necessary for cumulus expansion, oocyte maturation and release (37, 38). Altered expression of these genes might result in EFS, but this remains to be determined. Recently, a novel homozygous mutation in luteinizing hormone/choriogonadotropin receptor (LHCGR) gene, c.1345G>A (p. Ala449Thr) in exon 11 has been reported in a case which resulted in GEFS (27).

Although there is no agreement of EFS prevention and treatment, a timely rescue trigger can lead to cycle yielding successful results in selected cases. Ndukwe et al. (39) suggested a “cure” for EFS by re-administering hCG, after having failed to obtain oocytes, with variable success reported in the literature with this rescue technique. Reichman et al. (40) proposed a potential preventative measure against iatrogenic EFS by quantitative measurement of serum b-hCG post-trigger. When serum b-hCG level post-trigger was <5 *mIU/ml*, the cycles were effectively rescued with a repeat hCG followed by an OPU 24 *hr* later, resulting in a clinical pregnancy rate of 41.46% and live birth rate of 39.02%. In GnRHa triggered IVF cycles, as reported in a large retrospective study by Blazquez et al. (41) in oocyte donors, when EFS was encountered, a rescue protocol employed yielded satisfactory results with embryo transfer performed in 7 cycles (7/10; 70%) achieving two pregnancies. In our study, of the 8 cycles of EFS rescued, embryos were available for transfer in (6/9; 66.7%) subjects which were replaced in a frozenthawed cycle and they resulted in three (3/6=50%) clinical pregnancies. However, three patients failed to deliver any embryos for transfer due to poor quality oocytes and embryos. Although the exact reason for the compromised outcome in these three subjects remains obscure, coasting up to 72 *hr* and post maturity might have played a role in jeopardizing the cycle success. Our findings still justify a timely salvage of the cycle yielding, instead of abandoning the cycle at the time of initial retrieval, given the expenditure of time and resources. However, patients should be adequately informed regarding the cycle outcome, the likelihood of obtaining poor quality oocytes/embryos and non-availability of embryos for transfer. However, with rescuing hCG trigger in PCOS and hyper-responders, one should be cautious of a potential risk of OHSS. Of the eight EFS cases rescued with hCG, two manifested with severe OHSS, despite all preventive measures to prevent OHSS, requiring hospitalization with paracentesis and pleural tapping.

The fourth subject was a lean PCOS, who was hyper stimulated with a dosage of 1,250 *IU* of FSH, with 11 dominant follicles ≥17 *mm* and 7 intermediate follicles of 14–16 *mm* on the day of trigger and peak E2=7665 *pg/ml*, was triggered with GnRHa and an OPU 35 *hr* later which resulted in EFS. Her 12 *hr* post-trigger LH and P4 values were 10.2 *IU/L* and 2.3 *ng/ml*, respectively. Introspection into this case revealed that on the day of trigger, patient had received antagonist in the late evening (8 *pm*) followed by GnRHa trigger at night (10 *pm*). The time interval between the last dose of GnRH antagonist and GnRHa trigger should be adequate and Fauser et al. (5) demonstrated that 12 *hr* is sufficient for the GnRHa to displace GnRH antagonist from the receptor sites and result in optimal surge of gonadotropins. Hence in the above case, since EFS resulted due to wrong timing, a repeat dose of GnRHa trigger (Triptorelin 0.2 *mg*) administered with OPU 35 *hr* later, yielded 10 oocytes with 4–8 CG1 frozen embryos. A frozen-embryo transfer was done later, culturing 4–8 CG1 embryos to blastocysts which resulted in an ongoing pregnancy. Moreover, although this patient had high peak E2 levels, yet didn’t manifest with any signs or symptoms of either moderate or severe OHSS, as the patient was safely re-triggered with GnRHa.

The prognosis following EFS occurrence varies, depending on its etiology and in most of the cases, there was just a sporadic event with good clinical outcomes (42), as reported in our study. However, some suggest that the occurrence of EFS would indicate a poor outcome in subsequent cycles (43). Coskun et al. (44) also reported poor outcomes after genuine EFS in young good responder patients, with a risk of recurrence in later IVF cycles of 15–20% (3, 42, 44). The risk factors for recurrence were advanced age, prolonged infertility, and poor ovarian response (3, 28, 42) and these patients should be counselled regarding their lower chances of pregnancy.

Strengths of the study were evaluation of the incidence, underlying etiopathophysiology and prevention of EFS following GnRha trigger in a homogeneous, selected population of PCOS. Additionally, the cycle outcome following rescue trigger was analyzed and further the potential preventive measures were discussed. Limitations were the retrospective design and small sample size. Alternative study designs could be the comparison of cycles triggered with GnRHa versus hCG; however, in view of the vast evidence in terms of safety and comfort of GnRHa trigger in PCOS, it would be unethical to ensue such a study, only for scientific purposes. 12 *hr* post-trigger LH and P4 values were not available in all cases and based on the values, no intervention was undertaken. The study did not manifest the levels of post-trigger values predictive of EFS as the number of cases is too small and further larger studies are needed to be performed.

## Conclusion

Our experience with these EFS cases following GnRHa in PCOS, albeit small, given the rarity of its occurrence, suggests that majority of EFS are of false forms. Given the present findings, our results further demonstrate that these cases can be effectively salvaged which results in reasonably favorable outcome without wasting the treatment cycles. However, patients should be adequately informed regarding the overall likelihood of cycle failure.
